# Linkage of the California Pesticide Use Reporting Database with Spatial Land Use Data for Exposure Assessment

**DOI:** 10.1289/ehp.9518

**Published:** 2007-01-04

**Authors:** John R. Nuckols, Robert B. Gunier, Philip Riggs, Ryan Miller, Peggy Reynolds, Mary H. Ward

**Affiliations:** 1 Department of Environmental and Radiological Health Sciences, Colorado State University, Fort Collins, Colorado, USA; 2 Northern California Cancer Center, Berkeley, California, USA; 3 U.S. Department of Agriculture, Animal Plant Health Inspection Service, Fort Collins, Colorado, USA; 4 Division of Cancer Epidemiology and Genetics, National Cancer Institute, National Institutes of Health, Department of Health and Human Services, Bethesda, Maryland, USA

**Keywords:** agricultural land use, California, exposure assessment, GIS, pesticide use reporting

## Abstract

**Background:**

The State of California maintains a comprehensive Pesticide Use Reporting Database (CPUR). The California Department of Water Resources (CDWR) maps all crops in agricultural counties in California about once every 5 years.

**Objective:**

We integrated crop maps with CPUR to more accurately locate where pesticides are applied and evaluated the effects for exposure assessment.

**Methods:**

We mapped 577 residences and used the CPUR and CDWR data to compute two exposure metrics based on putative pesticide use within a 500-m buffer. For the CPUR metric, we assigned pesticide exposure to the residence proportionally for all square-mile Sections that intersected the buffer. For the CDWR metric, we linked CPUR crop-specific pesticide use to crops mapped within the buffer and assigned pesticide exposure. We compared the metrics for six pesticides: simazine, trifluralin (herbicides), dicofol, propargite (insecticides), methyl bromide, and metam sodium (fumigants).

**Results:**

For all six pesticides we found good agreement (88–98%) as to whether the pesticide use was predicted. When we restricted the analysis to residences with reported pesticide use in Sections within 500 m, agreement was greatly reduced (35–58%). The CPUR metric estimates of pesticide use within 500 m were significantly higher than the CDWR metric for all six pesticides.

**Conclusions:**

Our findings may have important implications for exposure classification in epidemiologic studies of agricultural pesticide use using CPUR. There is a need to conduct environmental and biological measurements to ascertain which, if any, of these metrics best represent exposure.

In regions of intense agricultural production, adverse health effects from pesticide exposures have increasingly become an area of public concern. Occupational exposure to agricultural pesticides has been associated with diseases such as cancer, immune system disorders, adverse reproductive outcomes, developmental disorders, and neurologic disease (Keifer and Mahurin 1997; Zahm et al. 1997). Recent studies have demonstrated that levels of specific pesticides in residential house dust are associated with the proximity of the residence to crop production areas where those pesticides were applied (Fenske et al. 2002; Lu et al. 2000; Simcox et al. 1995; Ward et al. 2006).

Since 1990, the State of California has required full reporting of agricultural pesticide use, with an expressed objective of providing pesticide use data for health risk assessment [California Department of Pesticide Regulation (CDPR) 2000]. The California Pesticide Use Reporting (CPUR) data have been used as a surrogate for exposure in a number of environmental epidemiologic studies (Bell et al. 2001; Clary and Ritz 2003; Mills 1998; Reynolds et al. 2002, 2004, 2005a, 2005b; Ritz and Yu 2000; Rull et al. 2006). The reporting unit for the database is one Section of the Public Land Survey System (approximately 1.0 mi^2^ or 2.6 km^2^). One limitation of the CPUR database is that pesticide use is not linked to a specific field within a Section where the pesticide application occurred, thus prohibiting exposure metrics that consider pesticide drift within a Section. Depending on location of the participant residence, this limitation could preclude considering distances of < 1.0 mile (1,609 m) in an exposure metric based on proximity to pesticide use. Pesticide drift models suggest that most deposition occurs within a few hundred meters of the application site (Raupach et al. 2001; Teske et al. 2002). A recent study by Ward et al. (2006) found that increasing acreage of corn and soybean fields within 750 m of homes in Iowa was associated with significantly elevated odds of detection of agricultural herbicides in house dust compared with homes with no crops within 750 m. The CDPR has recognized this limitation of the database and the need for a consistent spatial identifier that links the pesticide application to a specific field or parcel. In January 2000 the CDPR instituted guidelines for collecting information so that reported pesticide applications also identify specific fields with the application (CDPR 2000). However, these guidelines have yet to be implemented across the state, and will not be available for retrospective studies with exposure windows before implementation.

Here we present results of a study to develop an exposure metric to improve the spatial resolution of the CPUR data so that proximity to pesticide use within the reporting unit of a Section can be included in exposure assessment. We evaluated the effect such an improvement would have in terms of exposure assessment for an epidemiologic study.

## Methods

**Figure 1:**
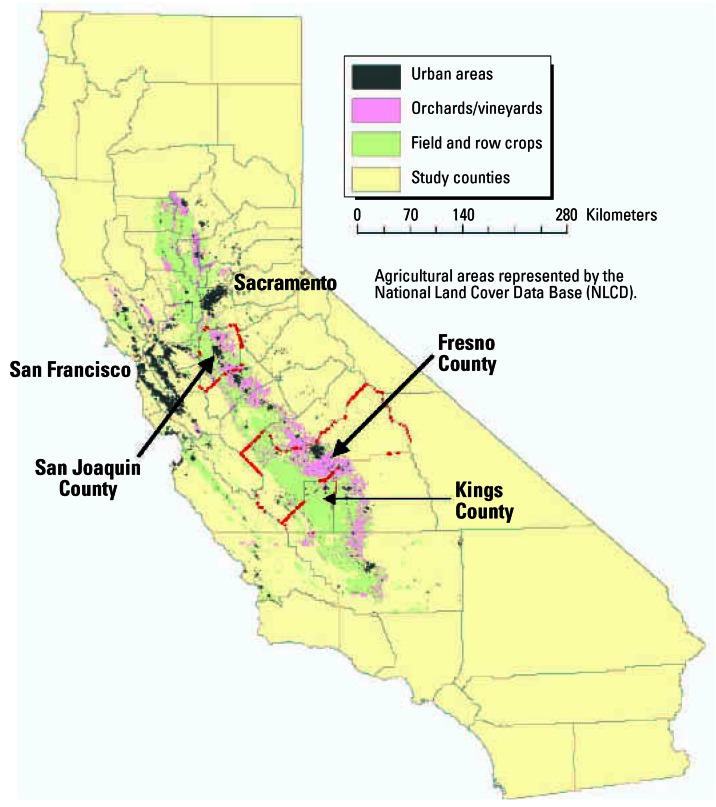
The three-county study area and general regional land use in California.

We conducted our study in three counties within the Central Valley of California (Figure 1). The Central Valley is one of the major agricultural production and pesticide use regions in the United States. We used three datasets: the CPUR pesticide database (CDPR 2000), crop maps from the California Department of Water Resources (CDWR), and residence locations from a childhood cancer study conducted by the California Department of Health Services (CDHS). Study participants provided informed consent according to procedures approved by the CDHS institutional review board. CPUR contains tabular information on agricultural and commercial nonagricultural pesticide applications. Only restricted-use pesticides were reported before 1990. In 1990, a full use reporting system was instituted that required applicators to report all agricultural pesticide use (CDPR 2000). The data are compiled annually at the county level and include information on the type and amount of pesticides applied, the date and method of application, and the crop treated. The geographic reporting unit for the database is a Meridian-Township-Range-Section (MTRS) in the United States Public Land Survey System. An MTRS, referred to as a Section, is a fixed-boundary parcel of land approximately 2.6 km^2^ (1.0 mi^2^) in area. The CPUR data used in our study were checked for likely errors (outliers) with respect to high application rates and corrected using the method reported by Gunier et al. (2001).

The CDWR is a state agency that surveys agricultural lands and crops for inventory mapping and analysis of water use. The maps are currently available in geographic information system (GIS) format for intermittent years between 1976 and 2004 (CDWR 2007). They are currently available for 38 counties, and are updated in counties with high agricultural land use about every 5–7 years. Individual agricultural field boundaries are delineated from aerial photography and used as the mapping unit for crop type. The minimum mapping unit of the CDWR is 0.81 hectares (0.003 mi^2^). Field crews identify crops and other land cover types usually once between July and September. The CDWR land use classification scheme contains 83 different land cover classes, including approximately 68 specific crop types. The data are collected using a 100% ground verification procedure by highly trained personnel, which should result in minimal error (Hawkins T, CDWR, personal communication).

We used latitude and longitude coordinates from a subset of subject residences from a CDHS epidemiologic study as the centroids for the construction of buffers for proximity metrics in this study. A total of 577 were geocoded for our three-county study area. A CDWR crop map was available for each county during the period 1988–1994: San Joaquin (1988), Kings (1991), and Fresno (1994) (CDWR 2007).

**Figure 2:**
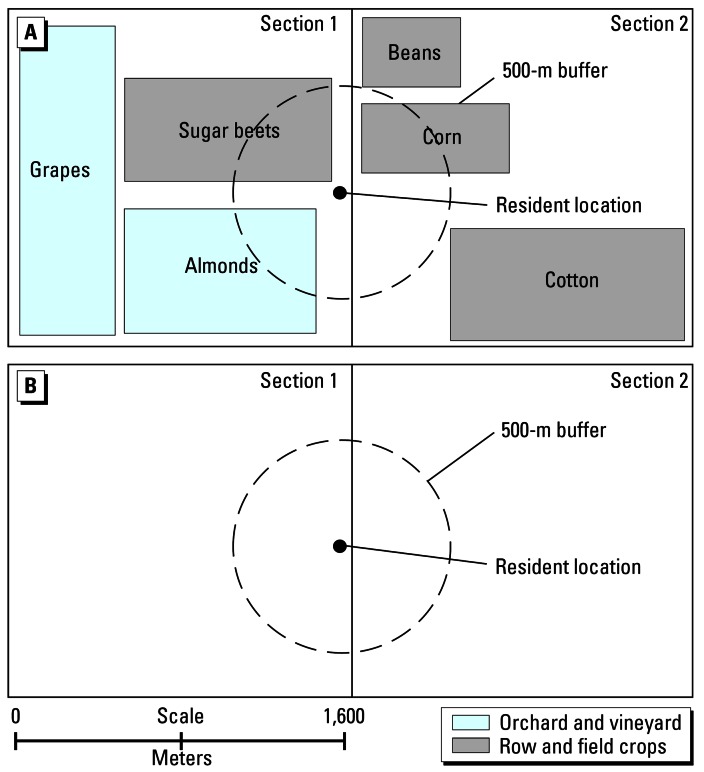
Comparison of **(***A*) a CDWR metric for a residence with a 500-m buffer intersecting two Sections and (*B*) a CPUR metric for a residence with a 500-m buffer intersecting two Sections.

In a GIS, we linked the CPUR crop-specific pesticide use for each Section in the three study counties to the CDWR crop maps for the corresponding Sections. We used a 500-m buffer (radius) around residences to define the zone of potential exposure from pesticide drift. This buffer distance was selected as an intermediate distance for the range of drift from pesticide applications (AgDRIFT Task Force 1997; Ward et al. 2000; Woods et al. 2001). We used the GIS to determine the area of crops within a 500-m radius (hereafter called 500-m buffer) around the residences. We computed a crop-specific application rate for each Section that intersected the 500-m buffer by dividing the annual pounds of a pesticide applied to the crop by the total area of the crop in the Section. We then multiplied the Section application rate by the crop area within the buffer to compute a CDWR-based pesticide exposure metric (Figure 2A) for each residence as follows:


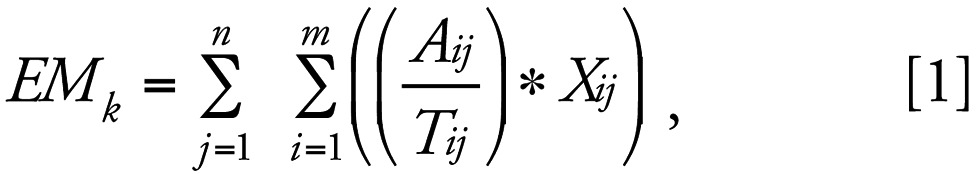


where *EM* is the exposure metric for a user-specified pesticide and residence, in pounds; *k* is the pesticide type (active ingredient); *i* is the crop type on which pesticide *k* was used in Sections *j* intersected by the 500-m buffer around the residence; *n* is the number of Sections intersected by the 500-m buffer around the residence; *m* is the total number of crop types on which pesticide *k* was applied in Sections *j; A_ij_* is the acreage of crop types *i* within Sections *j* and within 500 m of the residence; *T_ij_* is the total acreage of crop types *i* within Sections *j*; and *X_ij_* is the total annual pounds of pesticide *k* applied to crop types *i* within Sections *j*.

For a small percentage of Sections where CDWR indicated crops but there was no reported pesticide use in CPUR, we assumed no pesticide use on those crops.

We also computed a CPUR metric (Figure 2B) as follows:


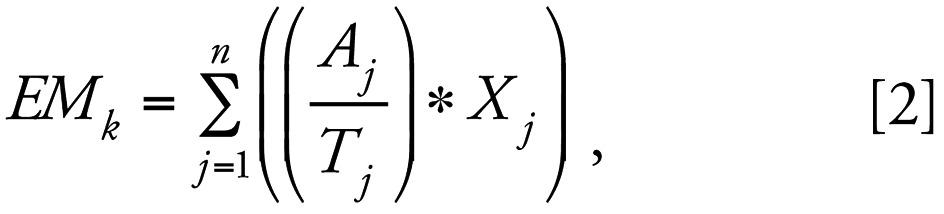


where *EM* is the exposure metric for a user-specified pesticide and residence, in pounds; *k* is the pesticide type (active ingredient) used in Sections *j* intersected by the 500-m buffer around the residence; *n* is the number of Sections intersected by the 500-m buffer around the residence; *A_i_* is the acreage of Sections *j* within 500 m of the residence; *T_i_* is the total acreage of Sections *j*; and *X_i_* is the total annual pounds of pesticide *k* applied within Sections *j.*

The primary difference between our two metrics is that the CDWR-based metric is a function of the acreage of crops on which the pesticide is used within the buffer, whereas the CPUR-based metric is a function of the total acreage of crops on which the pesticide is used within Section(s) intersected by the same buffer. For both methods, we divided the resulting pounds of pesticide use by the area of the buffer (0.31 mi^2^) to obtain the pesticide use density in pounds per square mile.

We calculated both exposure metrics for six pesticides with high use in the study area: simazine and trifluralin (herbicides), dicofol and propargite (insecticides), and methyl bromide and metam sodium (fumigants). To assess differences in exposure classification between the two metrics in the context of an epidemiologic study, we compared the metrics as dichotomous and as continuous variables.

We compared the two metrics’ classification of the 577 residences as exposed or unexposed with each pesticide and calculated percent agreement. We also calculated specificity of the CPUR metric compared with the CDWR metric as a gold standard (percent of homes classified as unexposed by CDWR metric that were classified as unexposed by CPUR). Because the CDWR metric depends on pesticide use reported in CPUR, sensitivity of the CPUR is 100% by definition. We calculated the specificity of CPUR for two exposure conditions: *a*) any pesticide use within the buffer, and *b*) ≥ 1 lb/mi^2^ within the buffer. The latter cut point was used by Reynolds et al. (2004, 2005b) to define the reference group in an epidemiologic study of childhood cancer and agricultural pesticide use. We calculated the prevalence of pesticide use based on the CDWR metric.

We computed the pounds of each pesticide per square mile of buffer predicted by each metric. We determined for each pesticide whether predictions for residences classified as “exposed” (i.e., ≥ 0 lb/mi^2^ applied within 500 m) were significantly different between the two metrics using the Wilcoxon signed-rank test (Ott and Longnecker 2001).

To examine the effect of classifying exposure using CPUR without crop maps, we categorized residential exposure predicted by the CPUR metric into quartiles, and then compared what proportion of residences fell into those categories using the CDWR metric. The cut point for the lowest quartile of pesticide use ranged from ≤ 4 lb/mi^2^ for trifluralin to ≤ 11 lb/mi^2^ for dicofol. The cut point for the highest quartile ranged from ≥ 48 lb/mi^2^ for trifluralin to ≥ 560 lb/mi^2^ for methyl bromide. We also calculated specificity and overall agreement between classification of exposure by the CDWR and CPUR metric for each pesticide, using the pesticide use above the 25th percentile of the CPUR metric to define the exposed population (e.g., 4 lbs/mi^2^ for trifluralin, as shown in Table 1). We conducted this analysis on the subset of homes with estimated pesticide use > 0 lb/mi^2^ within 500 m according to the CPUR metric.

## Results

Our comparison of the two metrics’ classification of residences as potentially exposed or unexposed (based on whether a specific pesticide use was predicted to be in the 500-m buffer) is presented in Table 2. Overall agreement ranged from 88% for triflurlalin to 97% for simazine. There was 98% agreement between the metrics for metam sodium, mostly because no use was reported in sections within 500 m of 504 (97%) of the residences. Classification was essentially the same when we compared the two metrics in predicting > 1.0 lb/mi^2^ application rates (not shown). When we excluded residences with no reported use of our study pesticides in Sections within 500 m, agreement between the two metrics was much lower. The number of participants in this subset averaged 105 (18% of the total study population) across all pesticides except metam sodium, which had a very low prevalence of use (0.2%) and was excluded from further analyses. Agreement for this subset averaged 50%, ranging from 35% for trifluralin to 58% for dicofol.

For any pesticide use within the 500-m buffer, the specificity of the CPUR metric ranged from 86% for propargite to 96% for dicofol (Table 3). The estimated prevalence of exposure (based on the CDWR metric) ranged from 5% for trifluralin and dicofol to 15% for propargite (Table 3). When we considered exposure as ≥ 1 lb/mi^2^ pesticide use within the buffer, specificity of the CPUR metric, and prevalence of exposure by the CDWR metric was essentially unchanged for each pesticide (data not shown).

The remainder of our analyses was based on the classification of residences using a quantitative exposure estimate (estimated pounds per square mile of pesticide use within 500 m). Our comparison of the two metrics for residences that were predicted to have any pesticide use within the 500-m buffer by either metric is presented in Table 1. The number of residences analyzed ranged from 48 for dicofol to 155 for propargite. The CPUR metric means (pounds per square mile) ranged from 30% to over 3-fold higher than the CDWR means. Likewise, there were typically large differences among the median, 25th, and 75th percentile values. There were statistically significant differences between predicted use (pounds per square mile) by the metrics for each pesticide analyzed (*p* < 0.001). When we restricted the analysis to those residences with ≥ 1.0 lb/mi^2^ applied within 500 m, the difference remained statistically significant for tri-fluralin (*p* = 0.020), propargite (*p* = 0.001), and dicofol (*p* = 0.017), but not for simazine (*p* = 0.093) and methyl bromide (*p* = 0.774).

**Figure 3:**
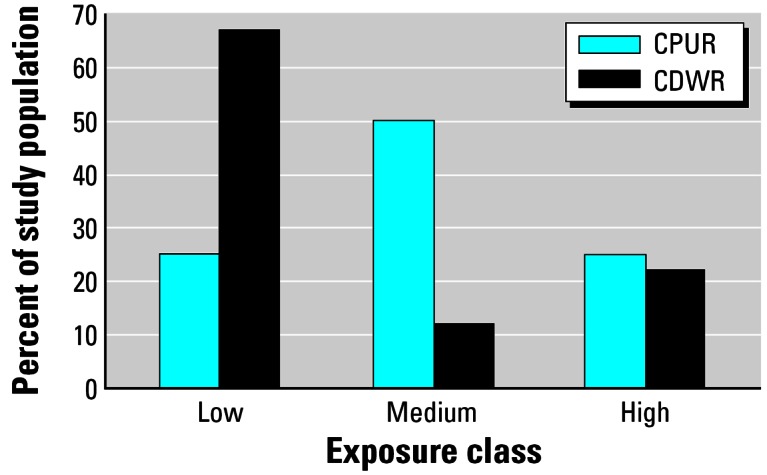
Average percent of residents classified as low (lowest quartile of CPUR lbs/mi^2^), medium (2nd and 3rd quartile), and high (4th quartile) exposure by the CPUR and CDWR metrics across the five pesticides analyzed.

When we compared what proportion of residences fell into quartiles of exposure predicted by the CPUR metric when the CDWR metric was applied, we observed a substantial shift from high or medium exposure to low (Figure 3). The number of CDWR-classified residences that fell into the lowest quartile category of the CPUR metric averaged 67%, ranging from 55% for propargite to 78% for methyl bromide. The same average for the highest CPUR quartile was 22% when classified using the CDWR metric, ranging from 8% for methyl bromide to 34% for simazine.

The results of our calculation of specificity and overall agreement between the two metrics when using the pesticide use above the 25th percentile of the CPUR metric to define the exposed population are presented in Table 4. Specificity ranged from 29% for methyl bromide to 45% for simazine and propargite. Overall agreement ranged from 45% for methyl bromide to 68% for propargite.

## Discussion

In this study, we demonstrated that CPUR data could be integrated with crop map data in a GIS to estimate the pesticide applications to specific crop fields within the reporting unit of CPUR (approximately 1 mi^2^). We computed a CDWR metric that estimates pesticide use within a 500-m (0.3 mi^2^) buffer around a residence. When we compared our CDWR metric with one based solely on CPUR pesticide use data, we found relatively good agreement in how the metrics assigned categorical exposure (pesticide use or no use in the buffer) for the six pesticides analyzed. However, further analysis indicated the high agreement was attributed mainly to the high proportion of the population with no reported use of the pesticides in Sections within 500 m of their residence. When this portion of the population is removed from the categorical analysis, the average agreement was reduced by about 46%. Thus, the CPUR metric classifies more residences as potentially exposed to pesticide applications than might be warranted based on the presence of crops with the specified pesticide use within 500 m of the residence.

When we compared the metrics’ predictions of quantitative pesticide use (pounds per square mile) for the same 577 residences, the CPUR metric consistently estimated significantly more pesticide use than did the CDWR metric, and the difference was statistically significant across all residences (*p* < 0.001). A large proportion of the population classified as having high exposure by the CPUR metric was classified into the low or medium exposure groups by the CDWR metric (Figure 3). As a result, specificity of the CPUR metric was low. These findings have important implications for epidemiologic studies using the CPUR database to estimate proximity of a study population to pesticide applications.

Specificity is the percent of the population who are correctly classified by the metric as unexposed (or in our case those in the lowest exposure category). In general, if misclassification of exposure is nondifferential between cases and controls, and the prevalence of exposure is low, small reductions in specificity of the exposure metric can result in substantial reductions in the risk estimate (Flegal et al. 1986; Kelsey et al. 1996; Nuckols et al. 2004; Rull and Ritz 2003).

Not surprisingly, our results indicate that a more spatially refined exposure metric based on the location of crops and their associated pesticide use in proximity to a residence (CDWR metric) dramatically reduces the estimated pesticide use near homes compared with a broader metric based on pesticide use in all Sections within 500 m of the residence (CPUR metric). Rull and Ritz (2003) also concluded that a metric based on CPUR alone overestimates the proximity of residences to pesticide use when compared with a metric based on CPUR data linked to CDWR crop maps. In their study, they mapped land use in broad categories of field crops, orchards, and vineyards, as opposed to determining specific crop types as we did. They computed sensitivity and specificity for a “broad” metric based on ever/never use of five pesticides in the Section with the residence and surrounding eight Sections, and for a “narrow” metric based on pesticide use only in the Section with the residence [a metric used previously by Bell et al. (2001)]. The prevalence of exposure based on the land use metric ranged from about 1–17%. The “broad metric” had 100% sensitivity and specificities ranging from 62 to 94% for the five pesticides evaluated, whereas the “narrow” metric had sensitivities ranging from 35 to 55% and specificities close to 99%. The resulting attenuation of odds ratios was substantially less for the “narrow” metric, further illustrating the importance of maximizing specificity when exposure prevalence is low. Rull and Ritz (2003) evaluated a set of agricultural pesticides different from ours; therefore we could not directly compare our metrics for estimating the prevalence of use, specificity, and sensitivity.

Further research is needed to determine whether the reported total pesticide use in a Section (PUR metric) or a metric based on location, acreage, and the associated pesticide use of crops in a Section better reflects residential exposure in the agricultural landscape of California. Studies that have measured pesticide deposition on passive samplers during aerial applications show that the deposition rate is related to the distance from the treated field (Richards et al. 2001; Woods et al. 2001). Woods et al. (2001) reported detection of endosulfan at distances of 500 m from cotton fields. Richards et al. (2001) reported detection of propanil at a distance of 138 m from the edge of rice fields. The maximum distance measured in that study of eight sites was 146 m, and included within home detection at two of the sites (distance = 108 and 103 m). Pesticide concentrations measured in house dust samples have also been associated with residential proximity to crops treated by ground spraying at distances of up to 400 m to an orchard (Fenske et al. 2002; Lu et al. 2000), and as a function of corn and soybean acreage in fields within 750 m (Ward et al. 2006). The results of a study that measured pesticide levels in indoor air and house dust suggest that a child may be exposed to a greater number of pesticides in the home by ingestion of house dust than by inhalation (Whitmore et al. 1994). Ambient air monitoring for agricultural pesticides has been conducted in agricultural communities of California during high pesticide use periods to assess general population exposures (Baker et al. 1997). A risk assessment based on outdoor air concentrations in California found significant noncancer and cancer risks even though monitoring sites were not located near field applications (Lee et al. 2002). A comparison of these same air monitoring data with CPUR data for several organophosphates showed that including agricultural pesticide use from Sections up to a 3-mile (4.8 km) radius from the monitoring site improved the correlation (Harnly et al. 2005).

Biological monitoring for organophosphate urinary metabolites has also been used to assess the relationship between exposure levels and proximity to crops, but the results have not been consistent. In one study (Lu et al. 2000), higher metabolite levels were found in the urine of children living within 200 feet (61 m) of treated orchards than in those living farther away. However, a similar study did not find significantly higher urine metabolite levels in children living in close proximity to treated orchards (Fenske et al. 2002). In a study of urinary pesticide metabolite levels in toddlers and residential proximity to treated fields in Imperial County, California, Royster et al. (2002) found no statistically significant differences in unadjusted or creatinine-adjusted median urinary dialkylphosphate concentrations of children living within 0.25 mile (402 m) or 0.5 mile (804 m) of the closest agricultural field when compared with the concentrations of those living > 0.25 or 0.5 mile from the closest field, respectively. A longitudinal study of children living in an agricultural community found higher levels of organophosphate metabolites in urine during the pesticide application months, but no statistically significant difference based on proximity (< 60 m vs. > 60 m) to treated fields (Koch et al. 2002). However, concentrations in urine of all children who resided within 60 m of an orchard were above the 50th percentile of the overall concentrations in the study population.

A limitation in the usefulness of our CDWR exposure metric for health studies is the number of years and geographic areas for which high-resolution crop maps exist for California. This limits the type of disease that can be studied in terms of incidence rates and latency periods. For example, in this study the residences represented birth addresses in three counties from 1983 to 1997 but our metrics were calculated using the one available year of crop maps for each county. In another study (Ward et al. 2000) we developed a crop pesticide exposure metric using satellite imagery that is available every year from the 1970s for most areas, which was useful in an area of large well-defined crop fields and limited crop species. However, for the time period of our study the complexities of using satellite imagery and remote sensing techniques to identify agricultural crops in California make it difficult for the CDWR to implement. As a result the CDWR has always performed land use surveys using photo interpretation of field boundaries and field visits (Hawkins T, CDWR, personal communication). Time and expense issues related to this method mean that a county-level survey can only be done every 5–7 years. Preliminary studies indicate that significant land cover changes can occur between these intervals, which could limit their utility in reconstructing location of pesticide use at the subsection level (Riggs PD, Nuckols JR, Buffler P, Ward MH, unpublished data).

In summary, we demonstrated that the CPUR data could be integrated in a GIS with crop maps to estimate pesticide exposure within a user-specified buffer around a residence. The pesticide use data in the CPUR database are quite detailed and go beyond most any other database in determining the location of pesticide use. However, our results indicate that residential pesticide use estimates differ greatly depending on the spatial scale at which exposure is estimated. If residential exposure is related to the amount of pesticides used only within 500–1,000 m of a residence, then a metric based on Section-level pesticide use is likely to considerably overestimate residential exposure. If residential exposure is associated with a greater distance or with a more complex relationship between distance, active ingredient, application method, and climatic conditions, then a metric relying primarily on a distance of 500 m may not be any more accurate in estimating exposure than a metric based on use in the entire Section. There is a clear need to evaluate other factors known to be associated with pesticide drift, such as wind speed and direction (Hewitt et al. 2002), in addition to the metrics we describe here. It is also clear that future research should include environmental and/or biological measurements in conjunction with a mapping study to ascertain which, if any, of these metrics best represent actual exposure to nearby residents. Such data would also define the optimal geographic extent (shape) an exposure metric should take to best estimate quantitative exposure for specific pesticides.

## Figures and Tables

**Table 1:** Comparison of CPUR and CDWR exposure metrics for residences (lb/mi^2^).^a^

Pesticide, metric	No.^a^	Mean	25th Percentile	Median	75th Percentile	Range
Trifluralin						
CDWR	30	14	< 1	< 1	5	< 1–204
CPUR	86	42	4	22	48	< 1–303
Simazine						
CDWR	68	42	< 1	< 1	33	< 1–525
CPUR	135	56	5	19	53	< 1–539
Propargite						
CDWR	88	86	< 1	1	60	< 1–1,417
CPUR	155	118	9	37	135	< 1–1,195
Dicofol						
CDWR	28	32	< 1	1	36	< 1–271
CPUR	48	60	11	37	82	< 1–326
Methyl bromide						
CDWR	50	162	< 1	< 1	6	< 1–7,613
CPUR	101	493	10	114	560	< 1–5,393

a Residences with > 0.0 lb/mi^2^ pesticide use within the 500-m buffer.

**Table 2:** Agreement between the CDWR and CPUR metrics for a dichotomous classification^a^ of pesticide use within 500 m of 577 residences in San Joaquin, Kings, and Fresno Counties.

Pesticide	Type	Overall agreement (%)	Agreement excluding residences with no reported use (%)
Trifluralin	Herbicide	90	35
Simazine	Herbicide	88	50
Propargite	Insecticide	88	56
Dicofol	Insecticide	97	58
Methyl bromide	Fumigant	91	50
Metam sodium	Fumigant	98	NA^b^

a CPUR: “exposed” if pesticide was applied in any Section within 500 m of residence; CDWR: “exposed” if pesticide was applied in any Section within 500 m of residence and a crop associated with use of that pesticide was located within the 500-m buffer.

b Not analyzed because of the low prevalence of use (0.2%) within 500 m of study residences.

**Table 3:** Specificity of the CPUR metric compared with CDWR metric for any pesticide use within 500 m of residence.^a^

Pesticide	Specificity (%)	Prevalence (%)
Trifluralin	90	5
Simazine	87	12
Propargite	86	15
Dicofol	96	5
Methyl bromide	90	9

a CPUR: “Yes” if use in any Section within 500 m of residence; CDWR: “Yes” if use in any Section within 500 m of residence and a crop associated with that pesticide use located within 500-m buffer.

**Table 4:** Specificity and percent agreement of the CPUR metric compared with the CDWR metric for residences where the CPUR metric was > 0.0 lb/mi^2^ within 500 m.

Pesticide^a^	No.	Specificity (%)	Agreement (%)
Trifluralin	86	38	56
Simazine	135	45	66
Propargite	155	45	68
Dicofol	48	38	56
Methyl bromide	101	29	45

a Exposure classified as > 25% percentile value of the CPUR metric (lb/mi ^2^). Sensitivity was 100% for all pesticides.

## References

[b1-ehp0115-000684] AgDRIFT Task Force 1997. A Summary of Ground Application Studies. Macon, MO:Stewart Agricultural Research Services. Available: http://www.agdrift.com [accessed 20 December 2006].

[b2-ehp0115-000684] Baker LW, Fitzell DL, Seiber JN, Parker TR, Shibamoto T, Poore MW (1997). Ambient air concentrations of pesticides in California. Environ Sci Technol.

[b3-ehp0115-000684] Bell EM, Hertz-Picciotto I, Beaumont J (2001). A case-control study of pesticides and fetal death due to congenital anomalies. Epidemiology.

[b4-ehp0115-000684] CDPR 2000. Pesticide Use Reporting: An Overview of California’s Unique Full Reporting System. Sacramento:California Department of Pesticide Regulation. Available: http://www.cdpr.ca.gov/docs/pur/purmain.htm [accessed 22 March 20007].

[b5-ehp0115-000684] CDWR (California Department of Water Resources) 2007. California Land and Water Use: Survey Data Access. Available: http://www.landwateruse.water.ca.gov/basicdata/landuse/landusesurvey.cfm [accessed 22 March 2007].

[b6-ehp0115-000684] Clary T, Ritz B (2003). Pancreatic cancer mortality and organo-chlorine pesticide exposure in California, 1989–1996. Am J Ind Med.

[b7-ehp0115-000684] Fenske RA, Lu C, Barr D, Needham L (2002). Children’s exposure to chlorpyrifos and parathion in an agricultural community in central Washington state. Environ Health Perspect.

[b8-ehp0115-000684] Flegal KM, Brownie C, Haas JD (1986). The effects of exposure misclassification on estimates of relative risks. Am J Epidemiol.

[b9-ehp0115-000684] Gunier RB, Harnly ME, Reynolds P, Hertz A, Von Behren J (2001). Agricultural pesticide use in California: pesticide prioritization, use densities, and population distributions for a childhood cancer study. Environ Health Perspect.

[b10-ehp0115-000684] Harnly M, McLaughlin R, Bradman A, Anderson M, Gunier R (2005). Correlating agricultural use of organophosphates with outdoor air concentrations: a particular concern for children. Environ Health Perspect.

[b11-ehp0115-000684] Hewitt AJ, Johnson DR, Fish JD, Hermansky CG, Valcore DL (2002). Development of the spray drift task force database for aerial applications. Environ Toxicol Chem.

[b12-ehp0115-000684] Keifer M Mahurin R 1997. Chronic neurological effects of pesticide overexposure. In: Occupational Medicine State of the Art Reviews: Human Health Effects of Pesticides (Keifer MC, ed). Philadelphia:Hanley and Belfus, 291–304. 9220487

[b13-ehp0115-000684] Kelsey JL Whittemore AS Evans AS Thompson WD 1996. Methods in Observational Epidemiology. 2nd ed. New York:Oxford University Press.

[b14-ehp0115-000684] Koch D, Lu C, Fisker-Andersen J, Jolley L, Fenske RA (2002). Temporal association of children’s pesticide exposure and agricultural spraying: report of a longitudinal biological monitoring study. Environ Health Perspect.

[b15-ehp0115-000684] Lee S, McLaughlin R, Harnly M, Gunier R, Kreutzer R (2002). Community exposures to airborne agricultural pesticides in California: ranking of inhalation risks. Environ Health Perspect.

[b16-ehp0115-000684] Lu C, Fenske RA, Simcox NJ, Kalman D (2000). Pesticide exposure of children in an agricultural community: evidence of household proximity to farmland and take home exposure pathways. Environ Research.

[b17-ehp0115-000684] Mills PK (1998). Correlation analysis of pesticide use data and cancer incidence rates in California counties. Arch Environ Health.

[b18-ehp0115-000684] Nuckols JR, Ward MH, Jarup L (2004). Using GIS for exposure assessment in environmental epidemiology studies. Environ Health Perspect.

[b19-ehp0115-000684] Ott L Longnecker MT 2001. An Introduction to Statistical Methods and Data Analysis. 5th ed. Pacific Grove, CA:Duxbury Publications.

[b20-ehp0115-000684] Raupach MR, Briggs PR, Ahmad N, Edge VE (2001). Endosulfan transport. II. Modeling airborne dispersal and deposition by spray and vapor. J Environ Qual.

[b21-ehp0115-000684] Reynolds P, Hurley SE, Goldberg DE, Yerabati S, Gunier RB, Hertz A (2004). Residential proximity to agricultural pesticide use and incidence of breast cancer in the California Teachers Study cohort. Environ Res.

[b22-ehp0115-000684] Reynolds P, Hurley SE, Gunier RB, Yerabati S, Quach T, Hertz A (2005a). Residential proximity to agricultural pesticide use and incidence of breast cancer in California, 1988–1997. Environ Health Perspect.

[b23-ehp0115-000684] Reynolds P, Von Behren J, Gunier RB, Goldberg DE, Harnly M, Hertz A (2005b). Agricultural pesticide use and childhood cancer in California. Epidemiology.

[b24-ehp0115-000684] Reynolds P, Von Behren J, Gunier RB, Goldberg DE, Hertz A, Harnly ME (2002). Childhood cancer and agricultural pesticide use: an ecologic study in California. Environ Health Perspect.

[b25-ehp0115-000684] Richards SM, McClure GYH, Lavy TL, Mattice JD, Keller RJ, Gand J (2001). Propanil (3,4-dichloropropionanilide). Particulate concentrations within and near the residences of families living adjacent to aerially sprayed rice fields. Arch Environ Contam Toxicol.

[b26-ehp0115-000684] Ritz B, Yu F (2000). Parkinson’s disease mortality and pesticide exposures in California 1984–1994. Int J Epidemiol.

[b27-ehp0115-000684] Royster MO, Hilborn ED, Barr D, Carty CL, Rhoney S, Walsh D (2002). A pilot study of global positioning system/geographical information system measurement of residential proximity to agricultural fields and urinary organophosphate metabolite concentrations in toddlers. J Expo Anal Environ Epidemiol.

[b28-ehp0115-000684] Rull R, Ritz B (2003). Historical pesticide exposure in California using pesticide use reports and land-use surveys: an assessment of misclassification error and bias. Environ Health Perspect.

[b29-ehp0115-000684] Rull R, Ritz B, Shaw GM (2006). Neural tube defects and maternal residential proximity to agricultural pesticide applications. Am J Epidemiol.

[b30-ehp0115-000684] Simcox NJ, Fenske RA, Wolz SA, Lee I, Kalman DA (1995). Pesticides in household dust and soil: exposure pathways for children of agricultural families. Environ Health Perspect.

[b31-ehp0115-000684] Teske ME, Bird SL, Esterly DM, Curbishley TB, Ray SL, Perry SG (2002). AgDRIFT: a model for estimating near-field spray drift from aerial applications. Environ Toxicol Chem.

[b32-ehp0115-000684] Ward MH, Lubin J, Giglierano J, Colt JS, Wolter C, Bekiroglu N (2006). Proximity to crops and residential exposure to agricultural pesticides in Iowa. Environ Health Perspect.

[b33-ehp0115-000684] Ward MH, Nuckols JR, Weigel SJ, Maxwell SK, Cantor KP, Miller RS (2000). Estimating environmental exposure to agricultural pesticides using remote sensing and a geographic information system. Environ Health Perspect.

[b34-ehp0115-000684] Whitmore RW, Immerman FW, Camann DE, Bond AE, Lewis RG, Schaum JL (1994). Non-occupational exposures to pesticides for residents of two U.S. cities. Arch Environ Contam Toxicol.

[b35-ehp0115-000684] Woods N, Craig IP, Dorr G, Young B (2001). Spray drift of pesticides arising from aerial application in cotton. J Environ Qual.

[b36-ehp0115-000684] Zahm SH, Ward MH, Blair A (1997). Pesticides and cancer. Occup Med - State of the Art Rev.

